# Sinus Pause and Cognitive Dysfunction: Diagnostic and Therapeutic Implications

**DOI:** 10.1002/ccr3.72940

**Published:** 2026-06-12

**Authors:** Mohd Aizuddin Mohd Zulastri, Muhammad Azlan Aseri, Syed Saleh Syed Sheikh, Imran Zainal Abidin

**Affiliations:** ^1^ Department of Cardiology University Malaya Medical Centre Kuala Lumpur Malaysia; ^2^ Department of Cardiology International Islamic University Malaysia Kuantan Malaysia

**Keywords:** bradyarrhythmia, cognitive decline, pacemaker, sinus node dysfunction

## Abstract

Bradyarrhythmia‐related cerebral hypoperfusion should be considered in elderly patients presenting with unexplained episodic cognitive decline, particularly when symptoms fluctuate or occur transiently. Prolonged rhythm monitoring may identify potentially treatable arrhythmic causes.

**Abbreviations**
ACC: American College of Cardiology
AHA: American Heart Association
ESC: European Society of Cardiology
HRS: Heart Rhythm Society
MRA: Magnetic resonance angiography
MRI: Magnetic resonance imaging

## 1 Introduction

Sinus node dysfunction may rarely present with cognitive decline due to recurrent cerebral hypoperfusion even without classical syncope. Pacemaker implantation may restore cerebral perfusion and reverse cognitive symptoms, highlighting the importance of considering bradyarrhythmia‐related hypoperfusion as a potentially reversible contributor to cognitive impairment.

Cognitive decline is an uncommon manifestation of sinus node dysfunction and may be mistaken for neurodegenerative or postoperative conditions. We report a 66 year‐old lady with atrial fibrillation and structural heart disease who presented with an atypical manifestation of tachy‐brady syndrome, in which progressive forgetfulness and transient inattention were the predominant symptoms rather than syncope or presyncope, with subsequent clinical improvement following pacemaker implantation and optimization of medical therapy.

## 2 Case History/Examination

A 66 year‐old female, previously worked as a hospital matron, with underlying atrial fibrillation (AF) and mixed rheumatic valvular disease—comprising severe mitral regurgitation, moderate mitral stenosis, severe tricuspid regurgitation, and moderate aortic regurgitation—underwent bioprosthetic mitral and aortic valve replacement, tricuspid annuloplasty, and left atrial appendage ligation in 2023. Her postoperative recovery was initially uneventful, and she remained clinically stable without angina or heart failure symptoms. The patient remained cognitively stable in the immediate postoperative period, with onset of symptoms occurring approximately 2 weeks after surgery. The patient subsequently developed both episodic and continuous cognitive symptoms, consisting of transient “blank spells” with loss of attention and progressive memory decline, which were a concern to both the patient and her family members. The episodic events were brief, lasting approximately 1–5 s, occurring 3–5 times per day, and were most noticeable at night during wakefulness. There was no clear association between symptoms and specific daily activities. There were no associated features of syncope, giddiness, automatisms, or postictal confusion. She denied chest pain or exercise limitation. Clinical examination was unremarkable; however, cognitive assessment revealed impaired short‐term memory and reduced attention. Objective assessment demonstrated impaired short‐term memory. Montreal Cognitive Assessment (MoCA) [1] score was 22/30, consistent with mild cognitive impairment although she remained independent in most activities of daily living. However, there was no baseline preoperative cognitive assessment available for comparison.

Her medication profile at the time of presentation, including indications and potential cognitive effects, is summarized in Table 1.

**TABLE 1:** Medication list at presentation.

Drug	Indication	Cognitive effect
Rivaroxaban 20 mg OD	Stroke prevention in AF	No direct cognitive impairment. May reduce cognitive decline by preventing thromboembolism.
Metoprolol 50 mg BD	Rate control in AF	Possible bradycardia and may cause fatigue, dizziness, and rare cognitive slowing in elderly.
Rosuvastatin 10 mg ON	Dyslipidaemia	Generally neutral. Rare reports of reversible memory disturbance.

## 3 Differential Diagnosis, Investigations and Treatment

The initial differential diagnoses included postoperative cognitive dysfunction, early neurodegenerative disease, vascular cognitive impairment, medication‐related cognitive effects, ictal bradycardia, and cerebral hypoperfusion related to arrhythmia.

Postoperative cognitive dysfunction was considered but was deemed less likely, as the patient was initially cognitively stable following surgery, with delayed onset of symptoms. Furthermore, the presence of both progressive and episodic inattention is not typical of postoperative cognitive dysfunction, which is generally more persistent [2]. Subclinical cerebral embolism following cardiac surgery was also considered but deemed less likely, as the patient remained adequately anticoagulated and neuroimaging showed no features of recent embolic infarction.

Ictal bradycardia syndrome was also considered; however, there were no clinical features suggestive of epilepsy, such as automatisms or postictal confusion [3]. EEG was not performed due to low clinical suspicion and the clear temporal correlation between symptoms and sinus pauses. The observed improvement following pacing—although not complete resolution—supports a primary cardiac mechanism, as pacing would not be expected to improve seizure‐related events.

Medication‐induced cognitive impairment was later excluded based on pharmacological review (Table 1). Although prior beta‐blocker therapy may have contributed to worsening bradycardia, significant sinus pauses persisted despite withholding metoprolol for 1 week prior to Holter monitoring, supporting underlying intrinsic sinus node dysfunction.

Atrial fibrillation is a well‐established cause of cognitive decline [4, 5]. The relative contribution of tachyarrhythmia versus bradyarrhythmia remains difficult to delineate.

A comprehensive laboratory evaluation was performed to exclude reversible causes of cognitive impairment, including relevant metabolic, nutritional, and infectious aetiologies. All results were within normal limits (Table 2).

**TABLE 2:** Laboratory investigations.

Test	Result	Lab Normal Range	Unit	Interpretation
Sodium	144	136–145	mmol/L	Normal
Potassium	4.3	3.6–5.2	mmol/L	Normal
Magnesium	0.88	0.53–1.11	mmol/L	Normal
Calcium (Corrected)	2.33	2.20–2.60	mmol/L	Normal
Phosphate	1.15	0.78–1.65	mmol/L	Normal
Urea	7.6	3.2–8.2	mmol/L	Normal
Creatinine	75	44–71	umol/L	Normal
Glucose	5.2	3.9–6.0 (fasting)	mmol/L	Normal
TSH	3.32	0.55–4.78	mIUl/L	Normal
Free T4	14.4	11.5–22.7	pmol/L	Normal
Vitamin B12	528	157–672	pmol/L	Normal
Folate	> 54.4	> 12.19	mmol/L	Normal
CRP	4.88	< 5.00	mg/L	Normal
Syphilis (Total antibodies to *Treponema Pallidum* )	Nonreactive	—	—	Negative
HIV‐1 & HIV‐2 antigen and antibodies	Nonreactive	—	—	Negative

A 72 h Holter monitoring (Figure 1) demonstrated 58 sinus pauses over 72 h (maximum duration 3.0 s), with temporal clustering and correlation with symptomatic episodes of inattention. Holter monitoring also demonstrated episodes of rapid atrial fibrillation, consistent with tachy‐brady syndrome. Although the longest pause was predominantly nocturnal (3.0 s), there was temporal clustering of pauses associated with symptomatic transient inattention during wakefulness, raising concern for clinically significant cerebral hypoperfusion. Further description of Holter monitoring was summarized in Table 3.

**FIGURE 1:**
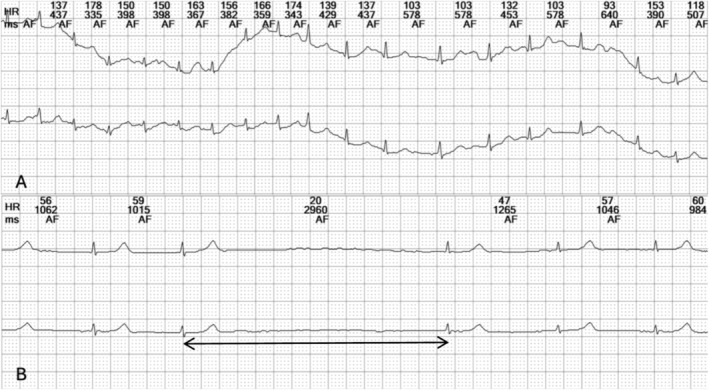
72 h HOLTER monitoring showed (A) atrial fibrillation with fast ventricular rate and (B) pause for 3.0 s (highlighted with arrow).

**TABLE 3:** Holter monitoring summary.

Parameter	Value	Additional details
Longest pause	3.0 s	Predominantly occurred during nocturnal period
Total number of pauses	58 episodes over 72 h	
Temporal distribution of pauses	Predominantly nocturnal and early morning	Clusters observed at early morning (0200–0400; 0700–0900) and late night (2100–2400)
Atrial fibrillation burden	100%	Persistent AF
Maximum heart rate	150 bpm	Occurred at 1700H
Minimum heart rate	34 bpm	Occurred at 0400H
Symptom correlation	Present	Episodes of inattention temporally associated with sinus pauses during awake state

Brain MRI and MRA (Figure 2) showed generalized cerebral atrophy and chronic small‐vessel disease.

**FIGURE 2:**
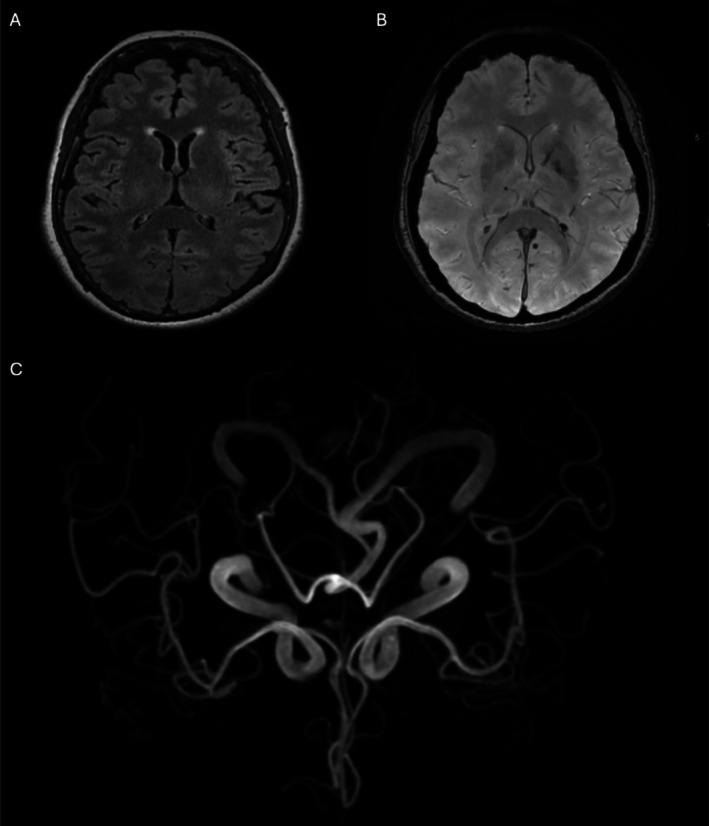
MRI and MRA Brain showed (A) diffuse volume loss of gyri in both hemispheres with widened sulci and global atrophy (B) multiple blooming artifacts seen at cerebral hemispheres, likely hemosiderin deposits, and (C) mild small vessel diseases.

Echocardiogram demonstrated preserved left ventricular ejection fraction (LVEF 62%), with normal functioning bioprosthetic mitral and aortic valves. There was bi‐atrial enlargement, and no intracardiac thrombus was identified.

In view of the suspected pathophysiology of significant sinus pauses causing cerebral hypoperfusion and contributing to her cognitive impairment, and to facilitate optimization of rate control for AF without exacerbating bradycardia/pauses, she underwent single‐chamber pacemaker implantation with a right ventricular lead positioned at the mid‐septum (Figure 3). The procedure was uncomplicated, and the device was programmed to rate‐responsive VVIR mode at 60 beats per minute. Her beta‐blocker therapy was subsequently up‐titrated to achieve improved rate control of AF.

**FIGURE 3:**
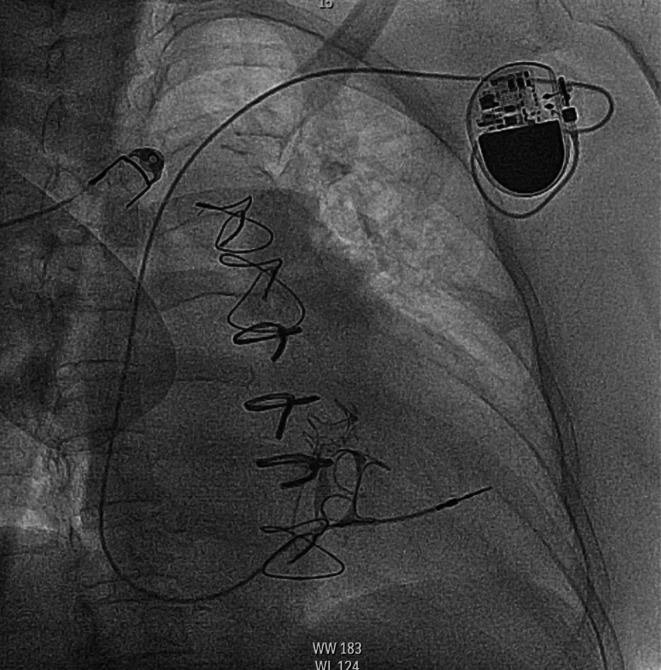
Chest X‐Ray showed post single chamber pacemaker insertion with tip of right ventricle lead in mid septum. Bioprosthetic valvular rings were also seen.

## 4 Conclusion and Results (Outcome and Follow‐Up)

At follow‐up, device interrogation revealed an 80% ventricular pacing burden, indicating frequent underlying pauses. After optimization of medical therapy, no further tachyarrhythmia episodes were recorded.

Cognitively, the patient reported substantial improvement, describing herself as almost back to her premorbid baseline.

This subjective improvement was supported by objective findings, with improvement in attention and memory domains on repeat MoCA assessment and overall score improved from 22/30 to 26/30.

She was subsequently comanaged with the geriatrics team and initiated on donepezil 2 weeks after pacemaker implantation, and enrolled in a structured cognitive enhancement program consisting of cognitive stimulation and cognitive activities, including reality orientation exercises, memory tasks, naming exercises, puzzles, and anagram‐based activities. Cognitive reassessment was performed 4 weeks after pacemaker implantation, demonstrating improvement in MoCA score from 22/30 to 26/30. As such, the relative contribution of pacemaker therapy and donepezil to cognitive improvement cannot be definitively determined.

## 5 Discussion

This case describes a patient with atrial fibrillation and structural heart disease who developed cognitive impairment with both continuous (progressive forgetfulness) and episodic (transient inattention) components, temporally associated with sinus pauses documented on Holter monitoring.

A comprehensive evaluation excluded metabolic, structural, and medication‐related causes of cognitive decline. Following pacemaker implantation, there was objective improvement in cognitive function (MoCA 22 to 26), supporting bradyarrhythmia‐related cerebral hypoperfusion as a contributing mechanism.

The same version of the MoCA was used for both baseline and follow‐up assessments. We acknowledge that improvement in MoCA scores may be influenced by a learning effect, particularly in individuals with higher educational attainment, particularly when the same version is repeated. Furthermore, the patient's background as a former hospital matron may increase familiarity with cognitive testing. However, the observed improvement was accompanied by clear subjective and functional recovery, supporting a clinically meaningful change beyond test familiarity. While the MoCA is a validated screening tool for global cognitive impairment, it is not highly sensitive to domain‐specific cognitive changes. Therefore, the observed improvement should be interpreted in conjunction with clinical and functional assessment rather than in isolation.

The observed cognitive improvement should be interpreted cautiously, as multiple therapeutic interventions occurred during the clinical course. Donepezil was initiated 2 weeks after pacemaker implantation, and reassessment was performed 2 weeks thereafter, making it difficult to isolate the independent contribution of each intervention, although donepezil generally demonstrates measurable clinical benefit over 12–24 weeks [6]. In addition, pacemaker implantation not only treated the documented sinus pauses but also enabled optimization of beta‐blocker therapy for rate control of atrial fibrillation without worsening bradycardia. Reduction in tachyarrhythmia burden may itself have contributed to improved cerebral perfusion and cognitive recovery. Therefore, the observed improvement was likely multifactorial rather than attributable solely to pacing therapy.

Donepezil also has been reported to be associated with bradyarrhythmia in rare cases [7]. However, in this patient, it was initiated after pacemaker implantation and therefore was unlikely to have contributed to the initial arrhythmic events.

Sick sinus syndrome is a frequent arrhythmia in older patients, most often presenting with bradycardia, pauses, or alternating tachy‐brady syndrome. Current ESC and AHA/ACC/HRS guidelines recommend pacemaker implantation in sinus node dysfunction only when symptoms such as syncope, presyncope, or heart failure are clearly attributable to bradycardia [8, 9]. Cognitive impairment on the other hand is not listed as an indication for pacemaker implantation. Atrial fibrillation is a well‐established cause of cognitive decline [5, 6]. This case uniquely highlights an atypical presentation of sinus node dysfunction with underlying atrial fibrillation, in which cognitive decline, rather than syncope, was the dominant symptom. The temporal association between pacing therapy and subsequent cognitive improvement supports bradyarrhythmia‐related cerebral hypoperfusion as a potentially contributing mechanism. Observational studies show that prolonged pauses with low cardiac output states accelerate white matter changes and cognitive decline in elderly patients [10]. Cerebral hypoperfusion is known to exacerbate both vascular dementia and Alzheimer's pathology, through impaired amyloid‐beta clearance and progressive white matter injury [11, 12]. Restoring perfusion with pacing may therefore help preserve cognition if introduced early. While cerebral hypoperfusion is a plausible mechanism linking bradyarrhythmia to cognitive dysfunction, direct evidence—such as cerebral blood flow assessment—was not available in this case. Therefore, the proposed mechanism remains inferential rather than directly demonstrated. Small studies have reported stabilization or improvement in cognitive scores following pacemaker implantation in patients with sinus node dysfunction [13]. However, large‐scale trials are lacking, and evidence remains limited. Arrhythmias of all types—including tachy‐ and bradyarrhythmia—are increasingly recognized as potentially modifiable contributors to cognitive decline risk, supporting closer integration of cardiac rhythm monitoring in patients with cognitive decline [14].

This report has several limitations. As a single case report, the findings are inherently limited in generalizability and should be interpreted as hypothesis‐generating rather than conclusive. In addition, the patient had multiple potential contributors to cognitive decline, including atrial fibrillation, structural valvular heart disease, and recent cardiac surgery, all of which may independently affect cognitive function. Furthermore, in the absence of a control group, it is not possible to attribute the observed cognitive improvement solely to pacemaker implantation.

Taken together, this case highlights a potential association between arrhythmia‐related cerebral hypoperfusion and cognitive dysfunction. Although it should not be interpreted as sole evidence to support modification of current guideline‐based indications for pacemaker therapy, the case emphasizes two key considerations. First, elderly patients presenting with unexplained or worsening cognitive impairment should undergo prolonged rhythm evaluation to exclude arrhythmia‐induced hypoperfusion. Second, current pacing indications may warrant reevaluation, with cognitive decline potentially serving as an adjunctive consideration rather than a primary indication.

## 6 Conclusion

This case highlights a potentially reversible and underrecognized cause of cognitive decline related to bradyarrhythmia‐induced cerebral hypoperfusion.

While pacemaker implantation was associated with improvement in cognitive function, complete symptom resolution was not observed, and causality cannot be definitively established. Careful exclusion of alternative diagnoses remains essential. These findings support the need for further studies to evaluate the role of rhythm control in cognitive outcomes. While cognitive improvement was observed, this was assessed primarily using a screening tool and should be interpreted alongside clinical findings. Further studies using comprehensive neurocognitive assessments are needed to better characterize this relationship.

## Author Contributions

**Mohd Aizuddin Mohd Zulastri:** conceptualization, project administration, writing – original draft, writing – review and editing. **Muhammad Azlan Aseri:** investigation, resources, writing – review and editing. **Syed Saleh Syed Sheikh:** investigation, resources, writing – review and editing. **Imran Zainal Abidin:** supervision, validation.

## Funding

The authors have nothing to report.

## Ethics Statement

Ethical approval for this case report was obtained from University Malaya Medical Centre Medical Research and Ethics Committee (MREC).

## Consent

Written informed consent for publication of the clinical details and clinical images was obtained from the patient. A copy of the consent form is available for review by the Editor of this journal.

## Conflicts of Interest

The authors declare no conflicts of interest.

## Data Availability

The data that support the findings of this study are available on request from the corresponding author. The data are not publicly available due to privacy or ethical restrictions.
